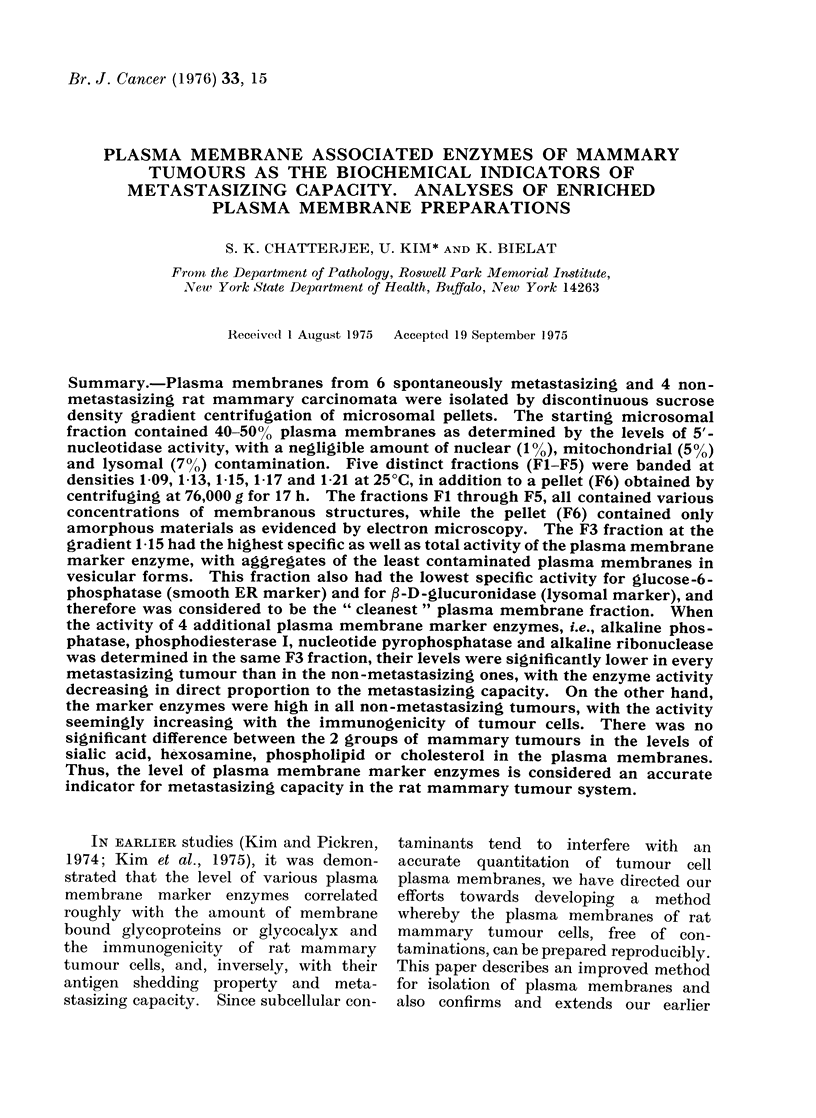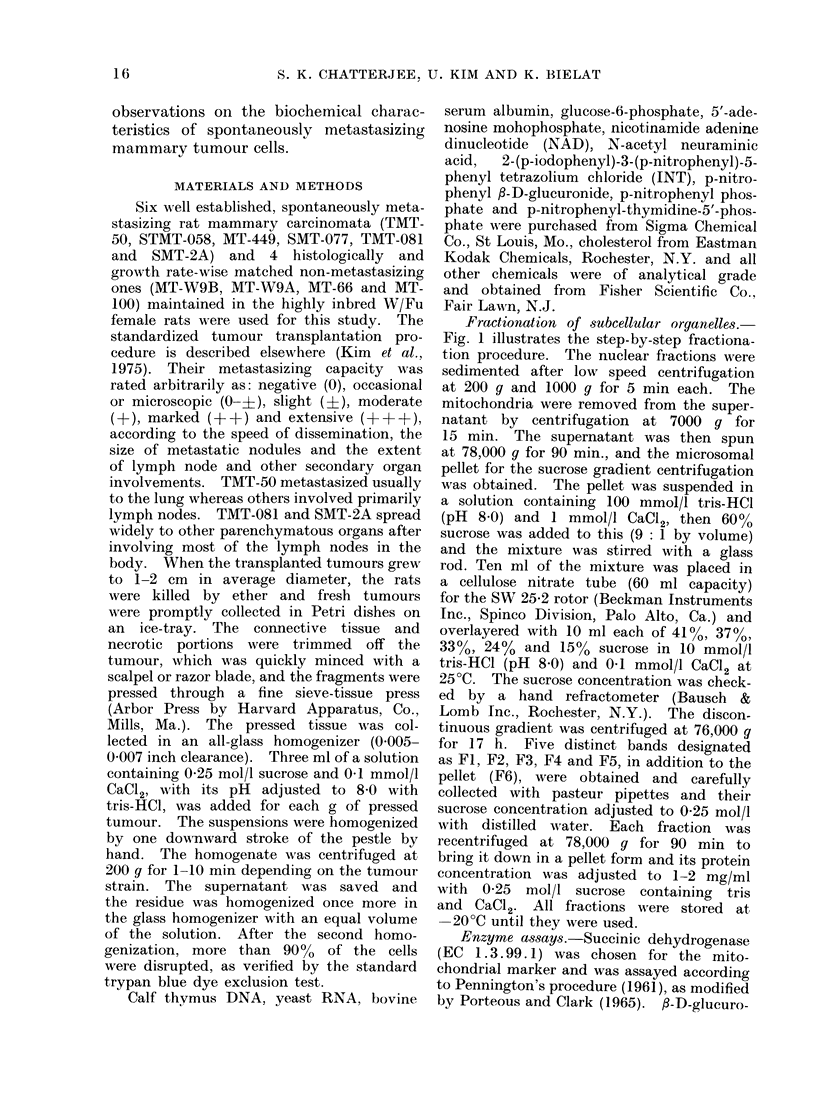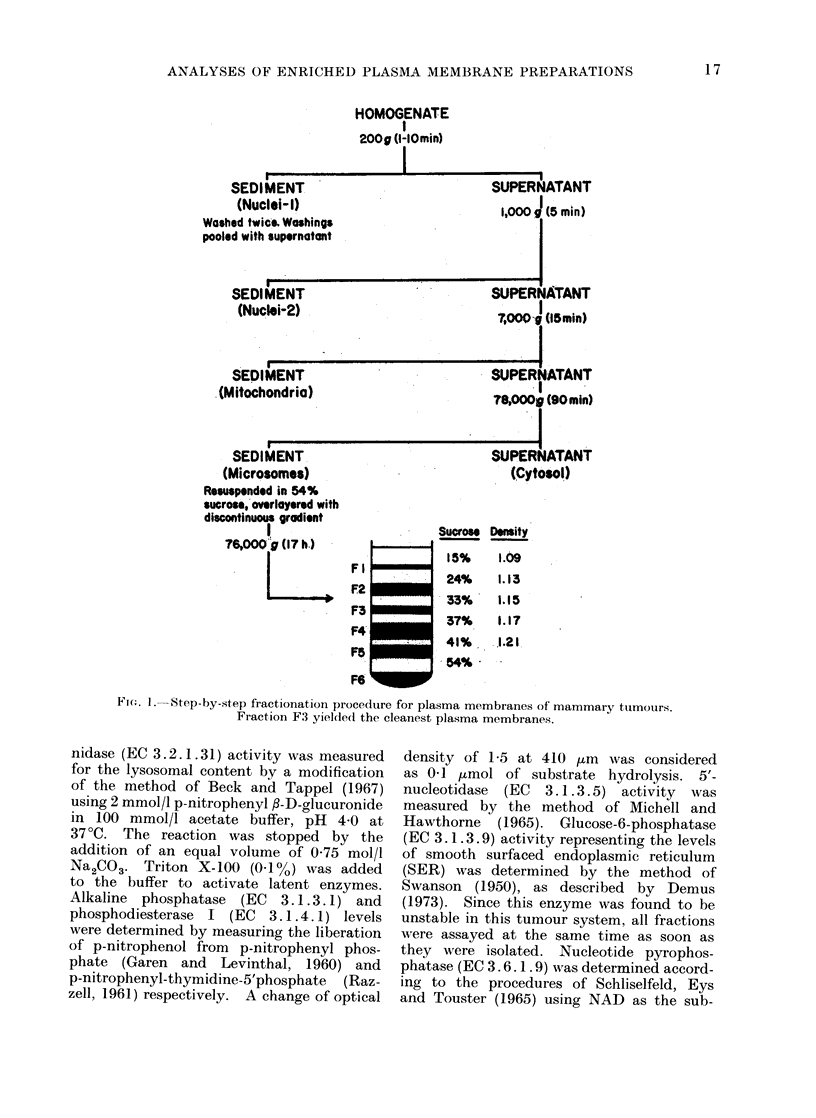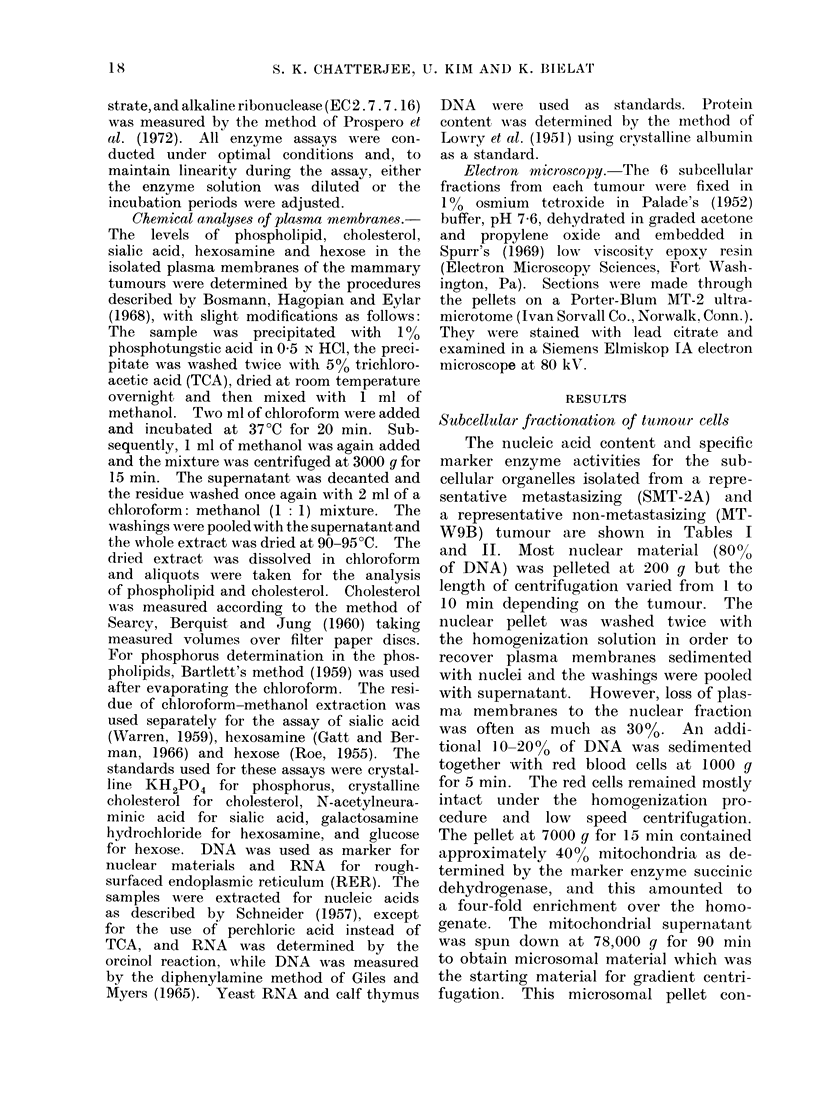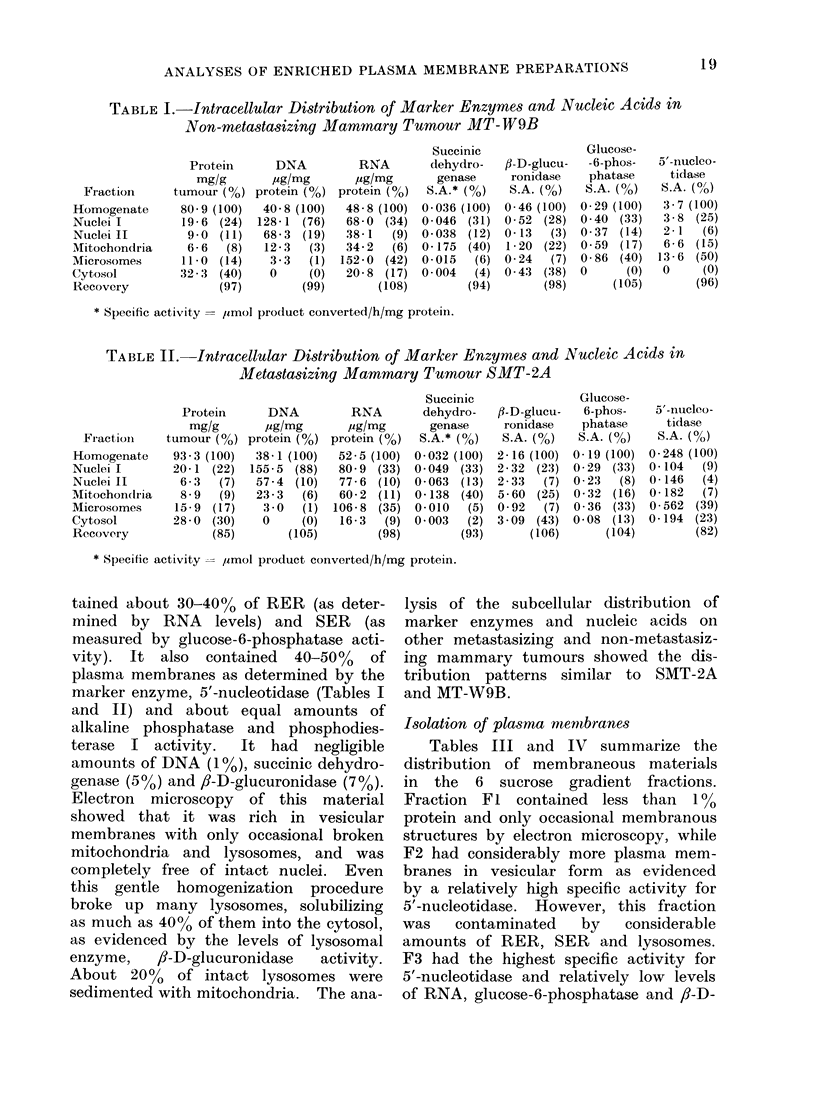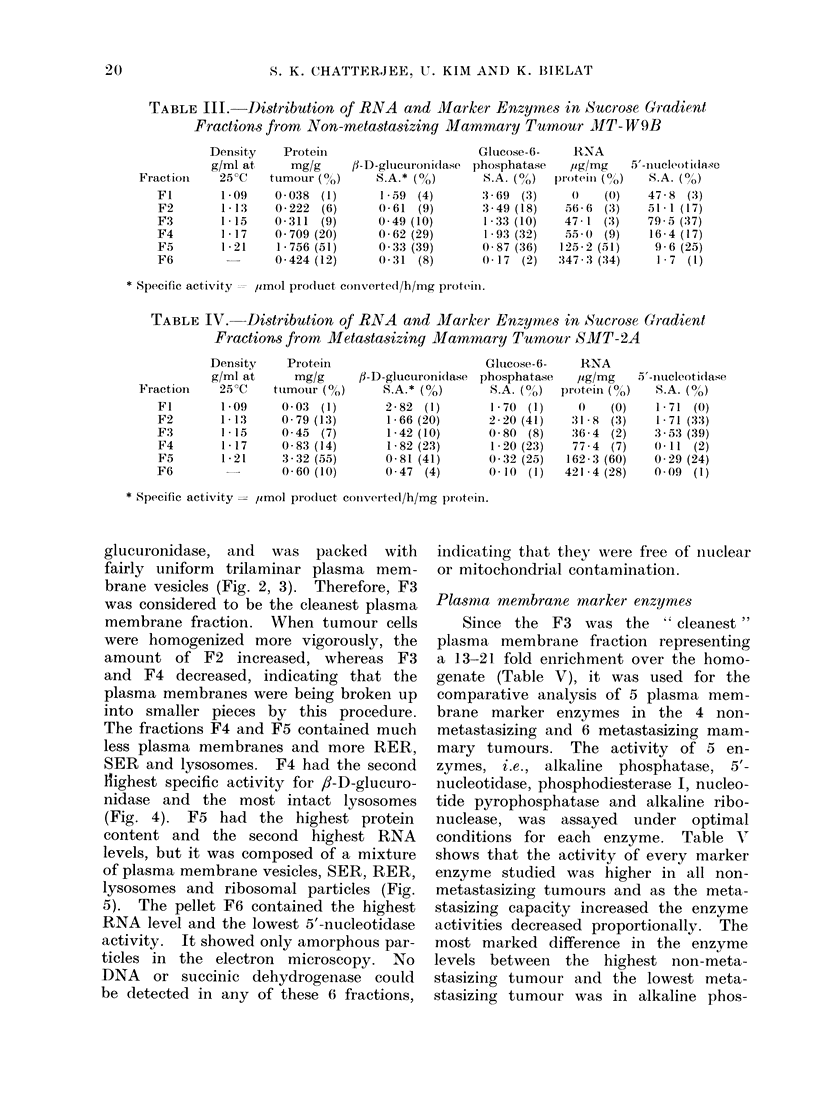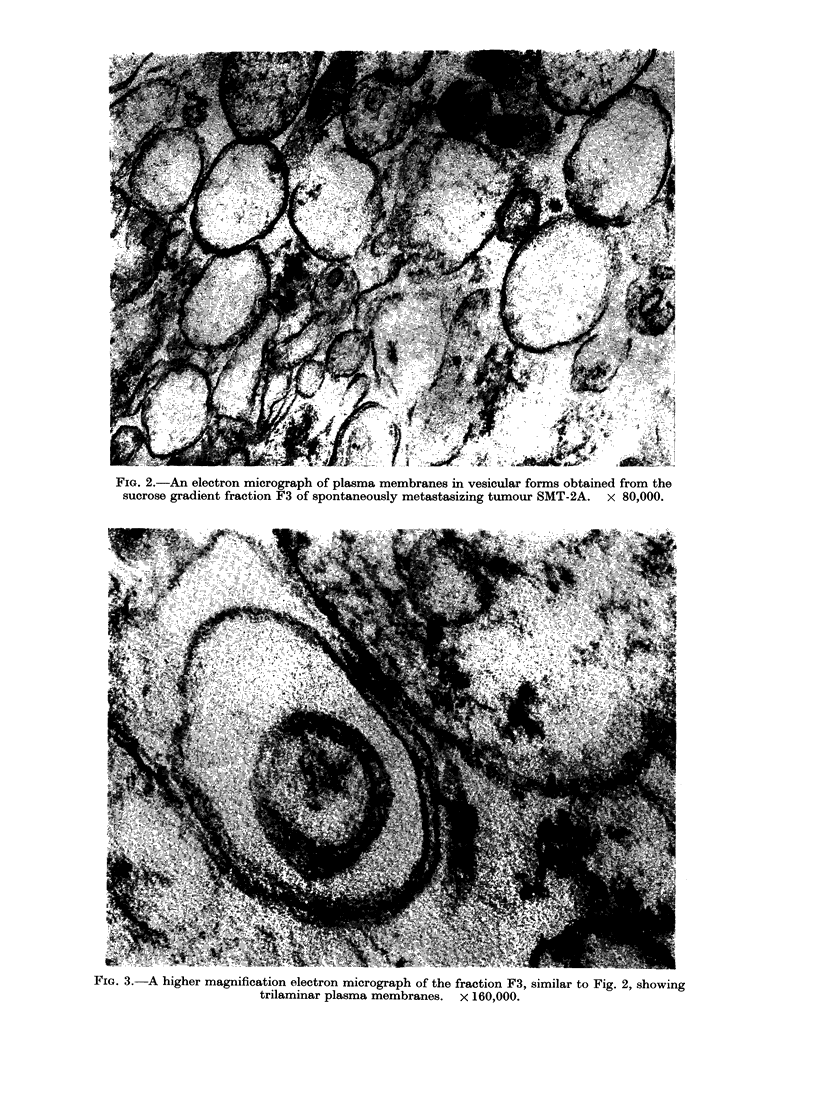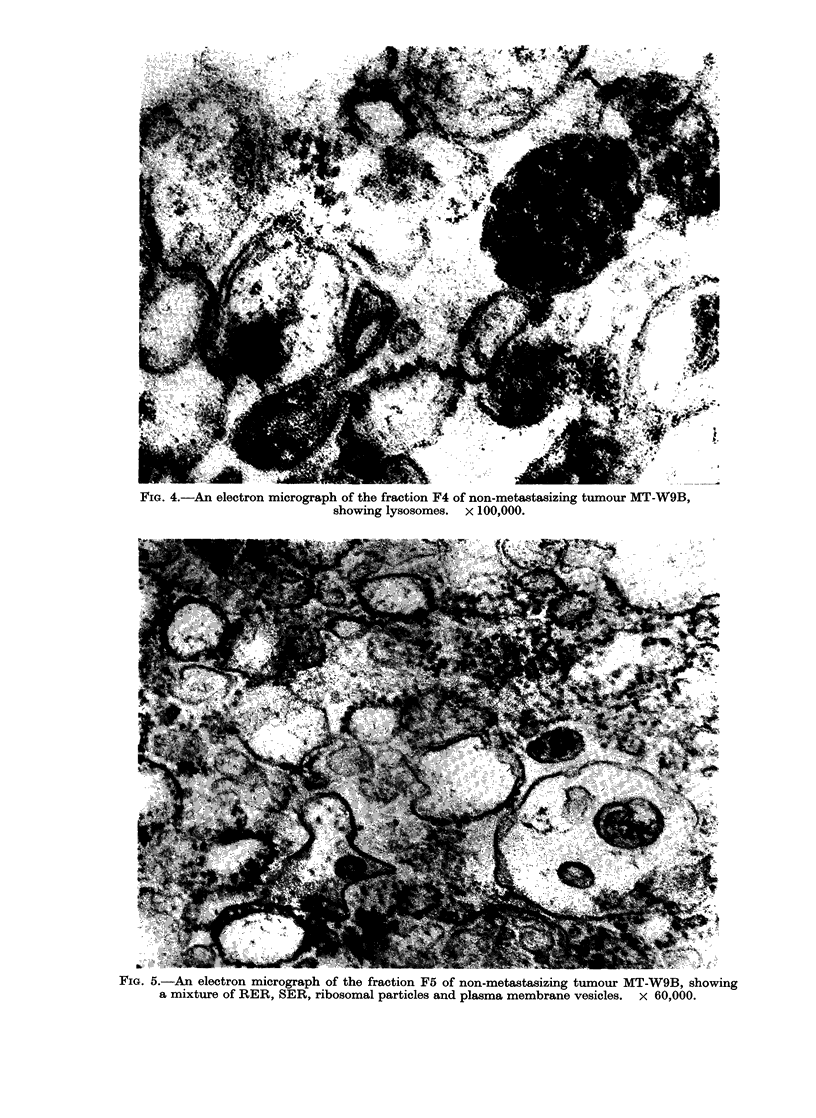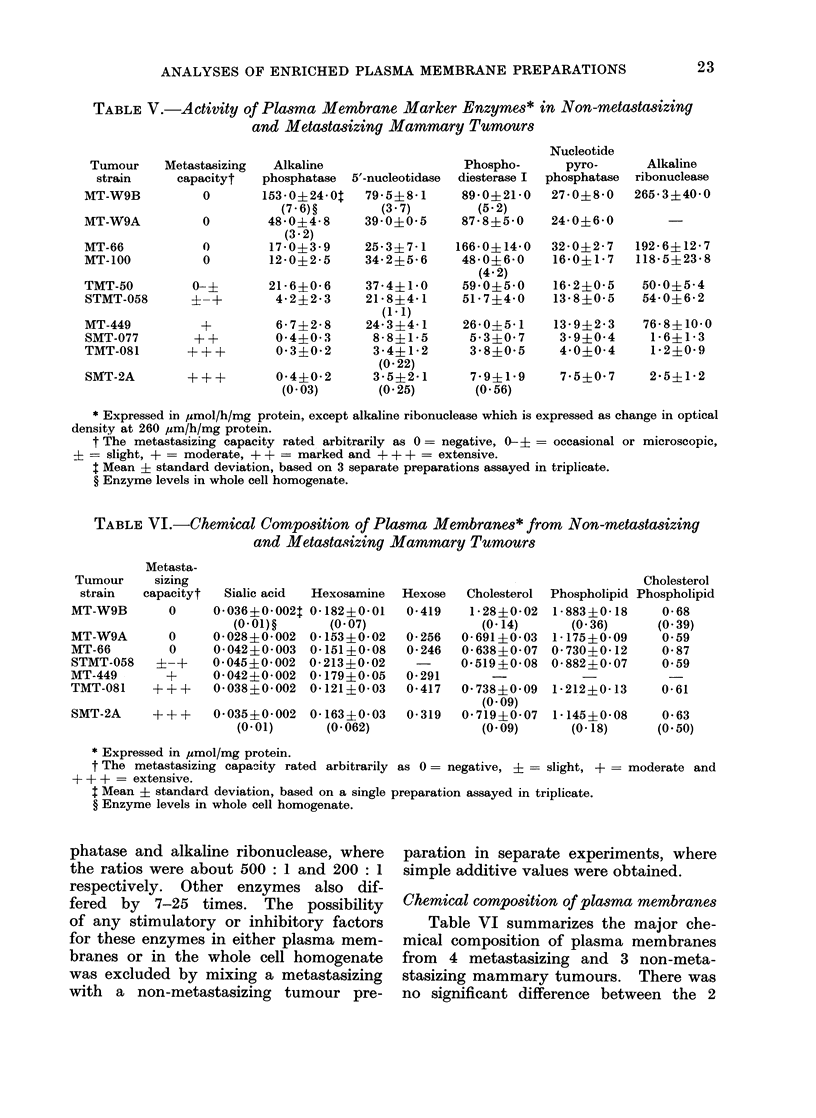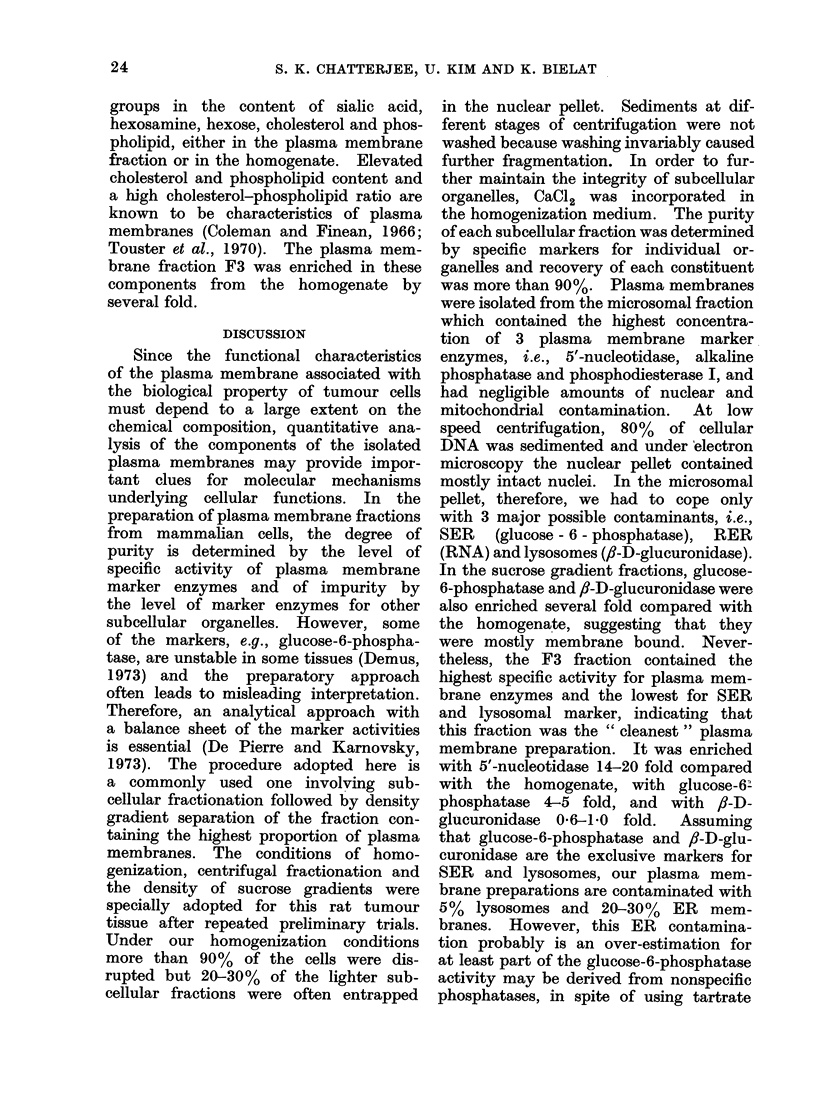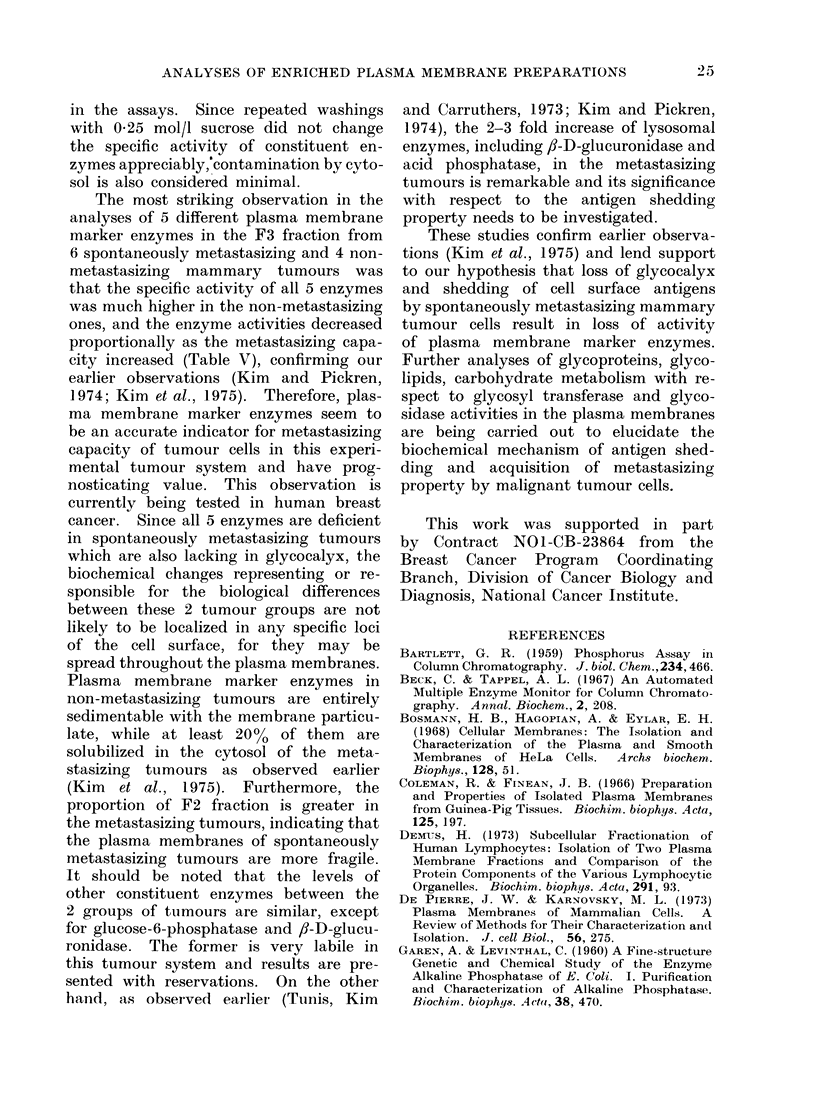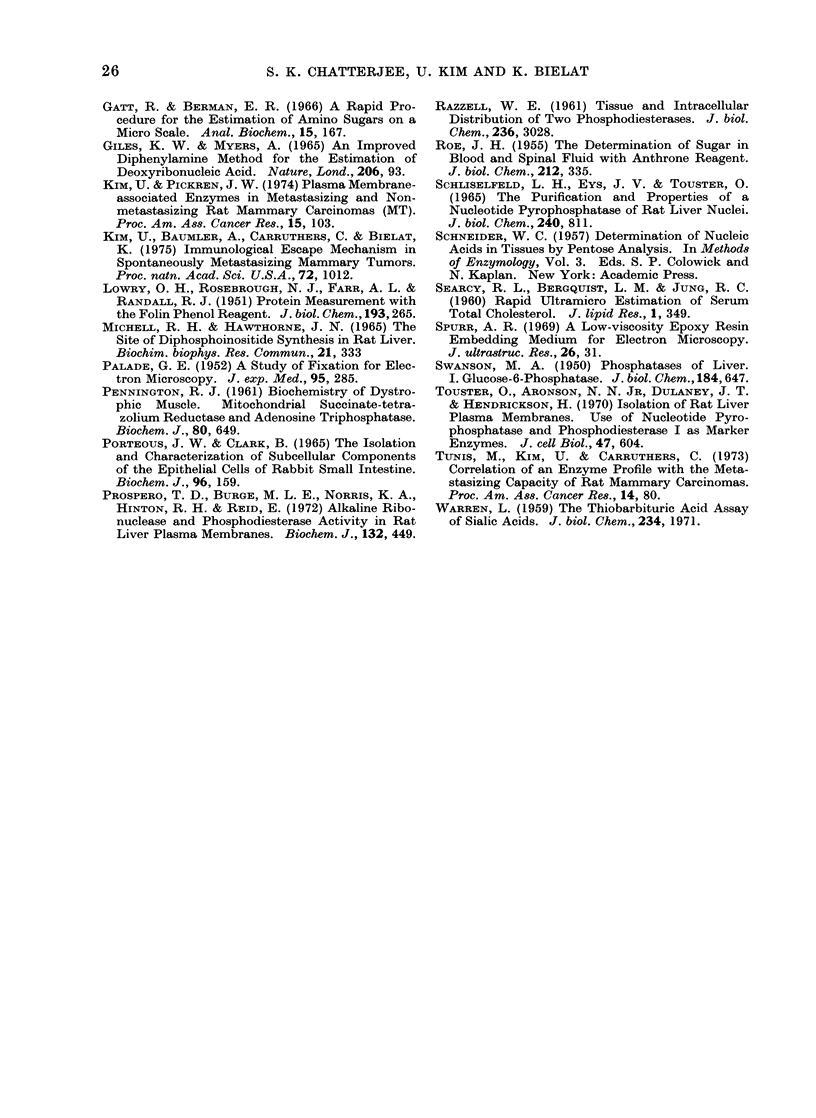# Plasma membrane associated enzymes of mammary tumours as the biochemical indicators of metastasizing capacity. Analyses of enriched plasma membrane preparations.

**DOI:** 10.1038/bjc.1976.3

**Published:** 1976-01

**Authors:** S. K. Chatterjee, U. Kim, K. Bielat

## Abstract

**Images:**


*Br. J. Cancer* (1976) **33**, 15

### PLASMA MEMBRANE ASSOCIATED ENZYMES OF MAMMARY TUMOURS AS THE BIOCHEMICAL INDICATORS OF METASTASIZING CAPACITY. ANALYSES OF ENRICHED PLASMA MEMBRANE PREPARATIONS

S. K. CHATTERJEE, U. KIM* AND K. BIELAT

*From the Department of Pathology, Roswell Park Memorial Institute, New York State Department of Health, Buffalo, New York* 14263

Received 1 August 1975 Accepted 19 September 1975

**Summary.—Plasma membranes from 6 spontaneously metastasizing and 4 non-metastasizing rat mammary carcinomata were isolated by discontinuous sucrose density gradient centrifugation of microsomal pellets. The starting microsomal fraction contained 40–50% plasma membranes as determined by the levels of 5′-nucleotidase activity, with a negligible amount of nuclear (1%), mitochondrial (5%) and lysomal (7%) contamination. Five distinct fractions (F1–F5) were banded at densities 1·09, 1·13, 1·15, 1·17 and 1·21 at 25°C, in addition to a pellet (F6) obtained by centrifuging at 76,000 *g* for 17 h. The fractions F1 through F5, all contained various concentrations of membranous structures, while the pellet (F6) contained only amorphous materials as evidenced by electron microscopy. The F3 fraction at the gradient 1·15 had the highest specific as well as total activity of the plasma membrane marker enzyme, with aggregates of the least contaminated plasma membranes in vesicular forms. This fraction also had the lowest specific activity for glucose-6-phosphatase (smooth ER marker) and for β-D-glucuronidase (lysomal marker), and therefore was considered to be the "cleanest" plasma membrane fraction. When the activity of 4 additional plasma membrane marker enzymes, *i.e.*, alkaline phosphatase, phosphodiesterase I, nucleotide pyrophosphatase and alkaline ribonuclease was determined in the same F3 fraction, their levels were significantly lower in every metastasizing tumour than in the non-metastasizing ones, with the enzyme activity decreasing in direct proportion to the metastasizing capacity. On the other hand, the marker enzymes were high in all non-metastasizing tumours, with the activity seemingly increasing with the immunogenicity of tumour cells. There was no significant difference between the 2 groups of mammary tumours in the levels of sialic acid, hexosamine, phospholipid or cholesterol in the plasma membranes. Thus, the level of plasma membrane marker enzymes is considered an accurate indicator for metastasizing capacity in the rat mammary tumour system.**

In EARLIER studies (Kim and Pickren, 1974; Kim *et al.*, 1975), it was demonstrated that the level of various plasma membrane marker enzymes correlated roughly with the amount of membrane bound glycoproteins or glycocalyx and the immunogenicity of rat mammary tumour cells, and, inversely, with their antigen shedding property and metastasizing capacity. Since subcellular contaminants tend to interfere with an accurate quantitation of tumour cell plasma membranes, we have directed our efforts towards developing a method whereby the plasma membranes of rat mammary tumour cells, free of contaminations, can be prepared reproducibly. This paper describes an improved method for isolation of plasma membranes and also confirms and extends our earlier observations on the biochemical characteristics of spontaneously metastasizing mammary tumour cells.

### MATERIALS AND METHODS

Six well established, spontaneously metastasizing rat mammary carcinomata (TMT-50, STMT-058, MT-449, SMT-077, TMT-081 and SMT-2A) and 4 histologically and growth rate-wise matched non-metastasizing ones (MT-W9B, MT-W9A, MT-66 and MT-100) maintained in the highly inbred W/Fu female rats were used for this study. The standardized tumour transplantation procedure is described elsewhere (Kim *et al.*, 1975). Their metastasizing capacity was rated arbitrarily as: negative (0), occasional or microscopic (0–±), slight (±), moderate (+), marked (++) and extensive (+++), according to the speed of dissemination, the size of metastatic nodules and the extent of lymph node and other secondary organ involvements. TMT-50 metastasized usually to the lung whereas others involved primarily lymph nodes. TMT-081 and SMT-2A spread widely to other parenchymatous organs after involving most of the lymph nodes in the body. When the transplanted tumours grew to 1–2 cm in average diameter, the rats were killed by ether and fresh tumours were promptly collected in Petri dishes on an ice-tray. The connective tissue and necrotic portions were trimmed off the tumour, which was quickly minced with a scalpel or razor blade, and the fragments were pressed through a fine sieve-tissue press (Arbor Press by Harvard Apparatus, Co., Mills, Ma.). The pressed tissue was collected in an all-glass homogenizer (0·005–0·007 inch clearance). Three ml of a solution containing 0·25 mol/l sucrose and 0·1 mmol/l $CaCl_2$, with its pH adjusted to 8·0 with tris-HCl, was added for each g of pressed tumour. The suspensions were homogenized by one downward stroke of the pestle by hand. The homogenate was centrifuged at 200 *g* for 1–10 min depending on the tumour strain. The supernatant was saved and the residue was homogenized once more in the glass homogenizer with an equal volume of the solution. After the second homogenization, more than 90% of the cells were disrupted, as verified by the standard trypan blue dye exclusion test.

Calf thymus DNA, yeast RNA, bovine serum albumin, glucose-6-phosphate, 5′-adenosine mohophosphate, nicotinamide adenine dinucleotide (NAD), N-acetyl neuraminic acid, 2-(p-iodophenyl)-3-(p-nitrophenyl)-5-phenyl tetrazolium chloride (INT), p-nitrophenyl β-D-glucuronide, p-nitrophenyl phosphate and p-nitrophenyl-thymidine-5′-phosphate were purchased from Sigma Chemical Co., St Louis, Mo., cholesterol from Eastman Kodak Chemicals, Rochester, N.Y. and all other chemicals were of analytical grade and obtained from Fisher Scientific Co., Fair Lawn, N.J.

*Fractionation of subcellular organelles.*—Fig. 1 illustrates the step-by-step fractionation procedure. The nuclear fractions were sedimented after low speed centrifugation at 200 *g* and 1000 *g* for 5 min each. The mitochondria were removed from the supernatant by centrifugation at 7000 *g* for 15 min. The supernatant was then spun at 78,000 *g* for 90 min., and the microsomal pellet for the sucrose gradient centrifugation was obtained. The pellet was suspended in a solution containing 100 mmol/l tris-HCl (pH 8·0) and 1 mmol/l $CaCl_2$, then 60% sucrose was added to this (9 : 1 by volume) and the mixture was stirred with a glass rod. Ten ml of the mixture was placed in a cellulose nitrate tube (60 ml capacity) for the SW 25·2 rotor (Beckman Instruments Inc., Spinco Division, Palo Alto, Ca.) and overlayered with 10 ml each of 41%, 37%, 33%, 24% and 15% sucrose in 10 mmol/l tris-HCl (pH 8·0) and 0·1 mmol/l $CaCl_2$ at 25°C. The sucrose concentration was checked by a hand refractometer (Bausch & Lomb Inc., Rochester, N.Y.). The discontinuous gradient was centrifuged at 76,000 *g* for 17 h. Five distinct bands designated as F1, F2, F3, F4 and F5, in addition to the pellet (F6), were obtained and carefully collected with pasteur pipettes and their sucrose concentration adjusted to 0·25 mol/l with distilled water. Each fraction was recentrifuged at 78,000 *g* for 90 min to bring it down in a pellet form and its protein concentration was adjusted to 1–2 mg/ml with 0·25 mol/l sucrose containing tris and $CaCl_2$. All fractions were stored at −20°C until they were used.

*Enzyme assays.*—Succinic dehydrogenase (EC 1.3.99.1) was chosen for the mitochondrial marker and was assayed according to Pennington's procedure (1961), as modified by Porteous and Clark (1965). β-D-glucuro-

FIG. 1.—Step-by-step fractionation procedure for plasma membranes of mammary tumours. Fraction F3 yielded the cleanest plasma membranes.

nidase (EC 3.2.1.31) activity was measured for the lysosomal content by a modification of the method of Beck and Tappel (1967) using 2 mmol/l p-nitrophenyl β-D-glucuronide in 100 mmol/l acetate buffer, pH 4·0 at 37 °C. The reaction was stopped by the addition of an equal volume of 0·75 mol/l $Na_2CO_3$. Triton X-100 (0·1%) was added to the buffer to activate latent enzymes. Alkaline phosphatase (EC 3.1.3.1) and phosphodiesterase I (EC 3.1.4.1) levels were determined by measuring the liberation of p-nitrophenol from p-nitrophenyl phosphate (Garen and Levinthal, 1960) and p-nitrophenyl-thymidine-5′phosphate (Razzell, 1961) respectively. A change of optical density of 1·5 at 410 μm was considered as 0·1 μmol of substrate hydrolysis. 5′-nucleotidase (EC 3.1.3.5) activity was measured by the method of Michell and Hawthorne (1965). Glucose-6-phosphatase (EC 3.1.3.9) activity representing the levels of smooth surfaced endoplasmic reticulum (SER) was determined by the method of Swanson (1950), as described by Demus (1973). Since this enzyme was found to be unstable in this tumour system, all fractions were assayed at the same time as soon as they were isolated. Nucleotide pyrophosphatase (EC 3.6.1.9) was determined according to the procedures of Schliselfeld, Eys and Touster (1965) using NAD as the substrate, and alkaline ribonuclease (EC2.7.7.16) was measured by the method of Prospero *et al.* (1972). All enzyme assays were conducted under optimal conditions and, to maintain linearity during the assay, either the enzyme solution was diluted or the incubation periods were adjusted.

*Chemical analyses of plasma membranes.*—The levels of phospholipid, cholesterol, sialic acid, hexosamine and hexose in the isolated plasma membranes of the mammary tumours were determined by the procedures described by Bosmann, Hagopian and Eylar (1968), with slight modifications as follows: The sample was precipitated with 1% phosphotungstic acid in 0·5 N HCl, the precipitate was washed twice with 5% trichloroacetic acid (TCA), dried at room temperature overnight and then mixed with 1 ml of methanol. Two ml of chloroform were added and incubated at 37°C for 20 min. Subsequently, 1 ml of methanol was again added and the mixture was centrifuged at 3000 *g* for 15 min. The supernatant was decanted and the residue washed once again with 2 ml of a chloroform: methanol (1 : 1) mixture. The washings were pooled with the supernatant and the whole extract was dried at 90–95°C. The dried extract was dissolved in chloroform and aliquots were taken for the analysis of phospholipid and cholesterol. Cholesterol was measured according to the method of Searcy, Berquist and Jung (1960) taking measured volumes over filter paper discs. For phosphorus determination in the phospholipids, Bartlett's method (1959) was used after evaporating the chloroform. The residue of chloroform–methanol extraction was used separately for the assay of sialic acid (Warren, 1959), hexosamine (Gatt and Berman, 1966) and hexose (Roe, 1955). The standards used for these assays were crystalline $KH_2PO_4$ for phosphorus, crystalline cholesterol for cholesterol, N-acetylneuraminic acid for sialic acid, galactosamine hydrochloride for hexosamine, and glucose for hexose. DNA was used as marker for nuclear materials and RNA for rough-surfaced endoplasmic reticulum (RER). The samples were extracted for nucleic acids as described by Schneider (1957), except for the use of perchloric acid instead of TCA, and RNA was determined by the orcinol reaction, while DNA was measured by the diphenylamine method of Giles and Myers (1965). Yeast RNA and calf thymus DNA were used as standards. Protein content was determined by the method of Lowry *et al.* (1951) using crystalline albumin as a standard.

*Electron microscopy.*—The 6 subcellular fractions from each tumour were fixed in 1% osmium tetroxide in Palade's (1952) buffer, pH 7·6, dehydrated in graded acetone and propylene oxide and embedded in Spurr's (1969) low viscosity epoxy resin (Electron Microscopy Sciences, Fort Washington, Pa). Sections were made through the pellets on a Porter-Blum MT-2 ultramicrotome (Ivan Sorvall Co., Norwalk, Conn.). They were stained with lead citrate and examined in a Siemens Elmiskop IA electron microscope at 80 kV.

### RESULTS

### *Subcellular fractionation of tumour cells*

The nucleic acid content and specific marker enzyme activities for the subcellular organelles isolated from a representative metastasizing (SMT-2A) and a representative non-metastasizing (MT-W9B) tumour are shown in Tables I and II. Most nuclear material (80% of DNA) was pelleted at 200 *g* but the length of centrifugation varied from 1 to 10 min depending on the tumour. The nuclear pellet was washed twice with the homogenization solution in order to recover plasma membranes sedimented with nuclei and the washings were pooled with supernatant. However, loss of plasma membranes to the nuclear fraction was often as much as 30%. An additional 10–20% of DNA was sedimented together with red blood cells at 1000 *g* for 5 min. The red cells remained mostly intact under the homogenization procedure and low speed centrifugation. The pellet at 7000 *g* for 15 min contained approximately 40% mitochondria as determined by the marker enzyme succinic dehydrogenase, and this amounted to a four-fold enrichment over the homogenate. The mitochondrial supernatant was spun down at 78,000 *g* for 90 min to obtain microsomal material which was the starting material for gradient centrifugation. This microsomal pellet con-

TABLE I.—*Intracellular Distribution of Marker Enzymes and Nucleic Acids in Non-metastasizing Mammary Tumour MT-W9B*

| Fraction | Protein mg/g tumour (%) | DNA μg/mg protein (%) | RNA μg/mg protein (%) | Succinic dehydrogenase S.A.* (%) | β-D-glucuronidase S.A. (%) | Glucose-6-phosphatase S.A. (%) | 5′-nucleotidase S.A. (%) |
|---|---|---|---|---|---|---|---|
| Homogenate | 80·9 (100) | 40·8 (100) | 48·8 (100) | 0·036 (100) | 0·46 (100) | 0·29 (100) | 3·7 (100) |
| Nuclei I | 19·6 (24) | 128·1 (76) | 68·0 (34) | 0·046 (31) | 0·52 (28) | 0·40 (33) | 3·8 (25) |
| Nuclei II | 9·0 (11) | 68·3 (19) | 38·1 (9) | 0·038 (12) | 0·13 (3) | 0·37 (14) | 2·1 (6) |
| Mitochondria | 6·6 (8) | 12·3 (3) | 34·2 (6) | 0·175 (40) | 1·20 (22) | 0·59 (17) | 6·6 (15) |
| Microsomes | 11·0 (14) | 3·3 (1) | 152·0 (42) | 0·015 (6) | 0·24 (7) | 0·86 (40) | 13·6 (50) |
| Cytosol | 32·3 (40) | 0 (0) | 20·8 (17) | 0·004 (4) | 0·43 (38) | 0 (0) | 0 (0) |
| Recovery | (97) | (99) | (108) | (94) | (98) | (105) | (96) |

* Specific activity = μmol product converted/h/mg protein.

TABLE II.—*Intracellular Distribution of Marker Enzymes and Nucleic Acids in Metastasizing Mammary Tumour SMT-2A*

| Fraction | Protein mg/g tumour (%) | DNA μg/mg protein (%) | RNA μg/mg protein (%) | Succinic dehydrogenase S.A.* (%) | β-D-glucuronidase S.A. (%) | Glucose-6-phosphatase S.A. (%) | 5′-nucleotidase S.A. (%) |
|---|---|---|---|---|---|---|---|
| Homogenate | 93·3 (100) | 38·1 (100) | 52·5 (100) | 0·032 (100) | 2·16 (100) | 0·19 (100) | 0·248 (100) |
| Nuclei I | 20·1 (22) | 155·5 (88) | 80·9 (33) | 0·049 (33) | 2·32 (23) | 0·29 (33) | 0·104 (9) |
| Nuclei II | 6·3 (7) | 57·4 (10) | 77·6 (10) | 0·063 (13) | 2·33 (7) | 0·23 (8) | 0·146 (4) |
| Mitochondria | 8·9 (9) | 23·3 (6) | 60·2 (11) | 0·138 (40) | 5·60 (25) | 0·32 (16) | 0·182 (7) |
| Microsomes | 15·9 (17) | 3·0 (1) | 106·8 (35) | 0·010 (5) | 0·92 (7) | 0·36 (33) | 0·562 (39) |
| Cytosol | 28·0 (30) | 0 (0) | 16·3 (9) | 0·003 (2) | 3·09 (43) | 0·08 (13) | 0·194 (23) |
| Recovery | (85) | (105) | (98) | (93) | (106) | (104) | (82) |

* Specific activity = μmol product converted/h/mg protein.

tained about 30–40% of RER (as determined by RNA levels) and SER (as measured by glucose-6-phosphatase activity). It also contained 40–50% of plasma membranes as determined by the marker enzyme, 5′-nucleotidase (Tables I and II) and about equal amounts of alkaline phosphatase and phosphodiesterase I activity. It had negligible amounts of DNA (1%), succinic dehydrogenase (5%) and β-D-glucuronidase (7%). Electron microscopy of this material showed that it was rich in vesicular membranes with only occasional broken mitochondria and lysosomes, and was completely free of intact nuclei. Even this gentle homogenization procedure broke up many lysosomes, solubilizing as much as 40% of them into the cytosol, as evidenced by the levels of lysosomal enzyme, β-D-glucuronidase activity. About 20% of intact lysosomes were sedimented with mitochondria. The analysis of the subcellular distribution of marker enzymes and nucleic acids on other metastasizing and non-metastasizing mammary tumours showed the distribution patterns similar to SMT-2A and MT-W9B.

### *Isolation of plasma membranes*

Tables III and IV summarize the distribution of membraneous materials in the 6 sucrose gradient fractions. Fraction F1 contained less than 1% protein and only occasional membranous structures by electron microscopy, while F2 had considerably more plasma membranes in vesicular form as evidenced by a relatively high specific activity for 5′-nucleotidase. However, this fraction was contaminated by considerable amounts of RER, SER and lysosomes. F3 had the highest specific activity for 5′-nucleotidase and relatively low levels of RNA, glucose-6-phosphatase and β-D-

TABLE III.—*Distribution of RNA and Marker Enzymes in Sucrose Gradient Fractions from Non-metastasizing Mammary Tumour MT-W9B*

| Fraction | Density g/ml at 25°C | Protein mg/g tumour (%) | β-D-glucuronidase S.A.* (%) | Glucose-6-phosphatase S.A. (%) | RNA μg/mg protein (%) | 5′-nucleotidase S.A. (%) |
|---|---|---|---|---|---|---|
| F1 | 1·09 | 0·038 (1) | 1·59 (4) | 3·69 (3) | 0 (0) | 47·8 (3) |
| F2 | 1·13 | 0·222 (6) | 0·61 (9) | 3·49 (18) | 56·6 (3) | 51·1 (17) |
| F3 | 1·15 | 0·311 (9) | 0·49 (10) | 1·33 (10) | 47·1 (3) | 79·5 (37) |
| F4 | 1·17 | 0·709 (20) | 0·62 (29) | 1·93 (32) | 55·0 (9) | 16·4 (17) |
| F5 | 1·21 | 1·756 (51) | 0·33 (39) | 0·87 (36) | 125·2 (51) | 9·6 (25) |
| F6 | — | 0·424 (12) | 0·31 (8) | 0·17 (2) | 347·3 (34) | 1·7 (1) |

* Specific activity = μmol product converted/h/mg protein.

TABLE IV.—*Distribution of RNA and Marker Enzymes in Sucrose Gradient Fractions from Metastasizing Mammary Tumour SMT-2A*

| Fraction | Density g/ml at 25°C | Protein mg/g tumour (%) | β-D-glucuronidase S.A.* (%) | Glucose-6-phosphatase S.A. (%) | RNA μg/mg protein (%) | 5′-nucleotidase S.A. (%) |
|---|---|---|---|---|---|---|
| F1 | 1·09 | 0·03 (1) | 2·82 (1) | 1·70 (1) | 0 (0) | 1·71 (0) |
| F2 | 1·13 | 0·79 (13) | 1·66 (20) | 2·20 (41) | 31·8 (3) | 1·71 (33) |
| F3 | 1·15 | 0·45 (7) | 1·42 (10) | 0·80 (8) | 36·4 (2) | 3·53 (39) |
| F4 | 1·17 | 0·83 (14) | 1·82 (23) | 1·20 (23) | 77·4 (7) | 0·11 (2) |
| F5 | 1·21 | 3·32 (55) | 0·81 (41) | 0·32 (25) | 162·3 (60) | 0·29 (24) |
| F6 | — | 0·60 (10) | 0·47 (4) | 0·10 (1) | 421·4 (28) | 0·09 (1) |

* Specific activity = μmol product converted/h/mg protein.

glucuronidase, and was packed with fairly uniform trilaminar plasma membrane vesicles (Fig. 2, 3). Therefore, F3 was considered to be the cleanest plasma membrane fraction. When tumour cells were homogenized more vigorously, the amount of F2 increased, whereas F3 and F4 decreased, indicating that the plasma membranes were being broken up into smaller pieces by this procedure. The fractions F4 and F5 contained much less plasma membranes and more RER, SER and lysosomes. F4 had the second highest specific activity for β-D-glucuronidase and the most intact lysosomes (Fig. 4). F5 had the highest protein content and the second highest RNA levels, but it was composed of a mixture of plasma membrane vesicles, SER, RER, lysosomes and ribosomal particles (Fig. 5). The pellet F6 contained the highest RNA level and the lowest 5′-nucleotidase activity. It showed only amorphous particles in the electron microscopy. No DNA or succinic dehydrogenase could be detected in any of these 6 fractions, indicating that they were free of nuclear or mitochondrial contamination.

*Plasma membrane marker enzymes*

Since the F3 was the "cleanest" plasma membrane fraction representing a 13–21 fold enrichment over the homogenate (Table V), it was used for the comparative analysis of 5 plasma membrane marker enzymes in the 4 non-metastasizing and 6 metastasizing mammary tumours. The activity of 5 enzymes, *i.e.*, alkaline phosphatase, 5′-nucleotidase, phosphodiesterase I, nucleotide pyrophosphatase and alkaline ribonuclease, was assayed under optimal conditions for each enzyme. Table V shows that the activity of every marker enzyme studied was higher in all non-metastasizing tumours and as the metastasizing capacity increased the enzyme activities decreased proportionally. The most marked difference in the enzyme levels between the highest non-metastasizing tumour and the lowest metastasizing tumour was in alkaline phos-

FIG. 2.—An electron micrograph of plasma membranes in vesicular forms obtained from the sucrose gradient fraction F3 of spontaneously metastasizing tumour SMT-2A. × 80,000.

FIG. 3.—A higher magnification electron micrograph of the fraction F3, similar to Fig. 2, showing trilaminar plasma membranes. × 160,000.

FIG. 4.—An electron micrograph of the fraction F4 of non-metastasizing tumour MT-W9B, showing lysosomes. $\times$ 100,000.

FIG. 5.—An electron micrograph of the fraction F5 of non-metastasizing tumour MT-W9B, showing a mixture of RER, SER, ribosomal particles and plasma membrane vesicles. $\times$ 60,000.

TABLE V.—*Activity of Plasma Membrane Marker Enzymes* in Non-metastasizing and Metastasizing Mammary Tumours*

| Tumour strain | Metastasizing capacity† | Alkaline phosphatase | 5′-nucleotidase | Phospho-diesterase I | Nucleotide pyro-phosphatase | Alkaline ribonuclease |
|---|---|---|---|---|---|---|
| MT-W9B | 0 | 153·0±24·0‡ (7·6)§ | 79·5±8·1 (3·7) | 89·0±21·0 (5·2) | 27·0±8·0 | 265·3±40·0 |
| MT-W9A | 0 | 48·0±4·8 (3·2) | 39·0±0·5 | 87·8±5·0 | 24·0±6·0 | — |
| MT-66 | 0 | 17·0±3·9 | 25·3±7·1 | 166·0±14·0 | 32·0±2·7 | 192·6±12·7 |
| MT-100 | 0 | 12·0±2·5 | 34·2±5·6 | 48·0±6·0 (4·2) | 16·0±1·7 | 118·5±23·8 |
| TMT-50 | 0–± | 21·6±0·6 | 37·4±1·0 | 59·0±5·0 | 16·2±0·5 | 50·0±5·4 |
| STMT-058 | ±–+ | 4·2±2·3 | 21·8±4·1 (1·1) | 51·7±4·0 | 13·8±0·5 | 54·0±6·2 |
| MT-449 | + | 6·7±2·8 | 24·3±4·1 | 26·0±5·1 | 13·9±2·3 | 76·8±10·0 |
| SMT-077 | ++ | 0·4±0·3 | 8·8±1·5 | 5·3±0·7 | 3·9±0·4 | 1·6±1·3 |
| TMT-081 | +++ | 0·3±0·2 | 3·4±1·2 (0·22) | 3·8±0·5 | 4·0±0·4 | 1·2±0·9 |
| SMT-2A | +++ | 0·4±0·2 (0·03) | 3·5±2·1 (0·25) | 7·9±1·9 (0·56) | 7·5±0·7 | 2·5±1·2 |

* Expressed in μmol/h/mg protein, except alkaline ribonuclease which is expressed as change in optical density at 260 μm/h/mg protein.
† The metastasizing capacity rated arbitrarily as 0 = negative, 0–± = occasional or microscopic, ± = slight, + = moderate, ++ = marked and +++ = extensive.
‡ Mean ± standard deviation, based on 3 separate preparations assayed in triplicate.
§ Enzyme levels in whole cell homogenate.

TABLE VI.—*Chemical Composition of Plasma Membranes* from Non-metastasizing and Metastasizing Mammary Tumours*

| Tumour strain | Metasta-sizing capacity† | Sialic acid | Hexosamine | Hexose | Cholesterol | Phospholipid | Cholesterol Phospholipid |
|---|---|---|---|---|---|---|---|
| MT-W9B | 0 | 0·036±0·002‡ (0·01)§ | 0·182±0·01 (0·07) | 0·419 | 1·28±0·02 (0·14) | 1·883±0·18 (0·36) | 0·68 (0·39) |
| MT-W9A | 0 | 0·028±0·002 | 0·153±0·02 | 0·256 | 0·691±0·03 | 1·175±0·09 | 0·59 |
| MT-66 | 0 | 0·042±0·003 | 0·151±0·08 | 0·246 | 0·638±0·07 | 0·730±0·12 | 0·87 |
| STMT-058 | ±–+ | 0·045±0·002 | 0·213±0·02 | — | 0·519±0·08 | 0·882±0·07 | 0·59 |
| MT-449 | + | 0·042±0·002 | 0·179±0·05 | 0·291 | — | — | — |
| TMT-081 | +++ | 0·038±0·002 | 0·121±0·03 | 0·417 | 0·738±0·09 (0·09) | 1·212±0·13 | 0·61 |
| SMT-2A | +++ | 0·035±0·002 (0·01) | 0·163±0·03 (0·062) | 0·319 | 0·719±0·07 (0·09) | 1·145±0·08 (0·18) | 0·63 (0·50) |

* Expressed in μmol/mg protein.
† The metastasizing capacity rated arbitrarily as 0 = negative, ± = slight, + = moderate and +++ = extensive.
‡ Mean ± standard deviation, based on a single preparation assayed in triplicate.
§ Enzyme levels in whole cell homogenate.

phatase and alkaline ribonuclease, where the ratios were about 500 : 1 and 200 : 1 respectively. Other enzymes also differed by 7–25 times. The possibility of any stimulatory or inhibitory factors for these enzymes in either plasma membranes or in the whole cell homogenate was excluded by mixing a metastasizing with a non-metastasizing tumour preparation in separate experiments, where simple additive values were obtained.

*Chemical composition of plasma membranes*

Table VI summarizes the major chemical composition of plasma membranes from 4 metastasizing and 3 non-metastasizing mammary tumours. There was no significant difference between the 2

groups in the content of sialic acid, hexosamine, hexose, cholesterol and phospholipid, either in the plasma membrane fraction or in the homogenate. Elevated cholesterol and phospholipid content and a high cholesterol–phospholipid ratio are known to be characteristics of plasma membranes (Coleman and Finean, 1966; Touster *et al.*, 1970). The plasma membrane fraction F3 was enriched in these components from the homogenate by several fold.

### DISCUSSION

Since the functional characteristics of the plasma membrane associated with the biological property of tumour cells must depend to a large extent on the chemical composition, quantitative analysis of the components of the isolated plasma membranes may provide important clues for molecular mechanisms underlying cellular functions. In the preparation of plasma membrane fractions from mammalian cells, the degree of purity is determined by the level of specific activity of plasma membrane marker enzymes and of impurity by the level of marker enzymes for other subcellular organelles. However, some of the markers, *e.g.*, glucose-6-phosphatase, are unstable in some tissues (Demus, 1973) and the preparatory approach often leads to misleading interpretation. Therefore, an analytical approach with a balance sheet of the marker activities is essential (De Pierre and Karnovsky, 1973). The procedure adopted here is a commonly used one involving subcellular fractionation followed by density gradient separation of the fraction containing the highest proportion of plasma membranes. The conditions of homogenization, centrifugal fractionation and the density of sucrose gradients were specially adopted for this rat tumour tissue after repeated preliminary trials. Under our homogenization conditions more than 90% of the cells were disrupted but 20–30% of the lighter subcellular fractions were often entrapped in the nuclear pellet. Sediments at different stages of centrifugation were not washed because washing invariably caused further fragmentation. In order to further maintain the integrity of subcellular organelles, $CaCl_2$ was incorporated in the homogenization medium. The purity of each subcellular fraction was determined by specific markers for individual organelles and recovery of each constituent was more than 90%. Plasma membranes were isolated from the microsomal fraction which contained the highest concentration of 3 plasma membrane marker enzymes, *i.e.*, 5′-nucleotidase, alkaline phosphatase and phosphodiesterase I, and had negligible amounts of nuclear and mitochondrial contamination. At low speed centrifugation, 80% of cellular DNA was sedimented and under electron microscopy the nuclear pellet contained mostly intact nuclei. In the microsomal pellet, therefore, we had to cope only with 3 major possible contaminants, *i.e.*, SER (glucose - 6 - phosphatase), RER (RNA) and lysosomes (β-D-glucuronidase). In the sucrose gradient fractions, glucose-6-phosphatase and β-D-glucuronidase were also enriched several fold compared with the homogenate, suggesting that they were mostly membrane bound. Nevertheless, the F3 fraction contained the highest specific activity for plasma membrane enzymes and the lowest for SER and lysosomal marker, indicating that this fraction was the " cleanest " plasma membrane preparation. It was enriched with 5′-nucleotidase 14–20 fold compared with the homogenate, with glucose-6-phosphatase 4–5 fold, and with β-D-glucuronidase 0·6–1·0 fold. Assuming that glucose-6-phosphatase and β-D-glucuronidase are the exclusive markers for SER and lysosomes, our plasma membrane preparations are contaminated with 5% lysosomes and 20–30% ER membranes. However, this ER contamination probably is an over-estimation for at least part of the glucose-6-phosphatase activity may be derived from nonspecific phosphatases, in spite of using tartrate in the assays. Since repeated washings with 0·25 mol/l sucrose did not change the specific activity of constituent enzymes appreciably, contamination by cytosol is also considered minimal.

The most striking observation in the analyses of 5 different plasma membrane marker enzymes in the F3 fraction from 6 spontaneously metastasizing and 4 non-metastasizing mammary tumours was that the specific activity of all 5 enzymes was much higher in the non-metastasizing ones, and the enzyme activities decreased proportionally as the metastasizing capacity increased (Table V), confirming our earlier observations (Kim and Pickren, 1974; Kim *et al.*, 1975). Therefore, plasma membrane marker enzymes seem to be an accurate indicator for metastasizing capacity of tumour cells in this experimental tumour system and have prognosticating value. This observation is currently being tested in human breast cancer. Since all 5 enzymes are deficient in spontaneously metastasizing tumours which are also lacking in glycocalyx, the biochemical changes representing or responsible for the biological differences between these 2 tumour groups are not likely to be localized in any specific loci of the cell surface, for they may be spread throughout the plasma membranes. Plasma membrane marker enzymes in non-metastasizing tumours are entirely sedimentable with the membrane particulate, while at least 20% of them are solubilized in the cytosol of the metastasizing tumours as observed earlier (Kim *et al.*, 1975). Furthermore, the proportion of F2 fraction is greater in the metastasizing tumours, indicating that the plasma membranes of spontaneously metastasizing tumours are more fragile. It should be noted that the levels of other constituent enzymes between the 2 groups of tumours are similar, except for glucose-6-phosphatase and β-D-glucuronidase. The former is very labile in this tumour system and results are presented with reservations. On the other hand, as observed earlier (Tunis, Kim and Carruthers, 1973; Kim and Pickren, 1974), the 2–3 fold increase of lysosomal enzymes, including β-D-glucuronidase and acid phosphatase, in the metastasizing tumours is remarkable and its significance with respect to the antigen shedding property needs to be investigated.

These studies confirm earlier observations (Kim *et al.*, 1975) and lend support to our hypothesis that loss of glycocalyx and shedding of cell surface antigens by spontaneously metastasizing mammary tumour cells result in loss of activity of plasma membrane marker enzymes. Further analyses of glycoproteins, glycolipids, carbohydrate metabolism with respect to glycosyl transferase and glycosidase activities in the plasma membranes are being carried out to elucidate the biochemical mechanism of antigen shedding and acquisition of metastasizing property by malignant tumour cells.

This work was supported in part by Contract NO1-CB-23864 from the Breast Cancer Program Coordinating Branch, Division of Cancer Biology and Diagnosis, National Cancer Institute.

### REFERENCES

Bartlett, G. R. (1959) Phosphorus Assay in Column Chromatography. *J. biol. Chem.*, **234**, 466.

Beck, C. & Tappel, A. L. (1967) An Automated Multiple Enzyme Monitor for Column Chromatography. *Annal. Biochem.*, **2**, 208.

Bosmann, H. B., Hagopian, A. & Eylar, E. H. (1968) Cellular Membranes: The Isolation and Characterization of the Plasma and Smooth Membranes of HeLa Cells. *Archs biochem. Biophys.*, **128**, 51.

Coleman, R. & Finean, J. B. (1966) Preparation and Properties of Isolated Plasma Membranes from Guinea-Pig Tissues. *Biochim. biophys. Acta*, **125**, 197.

Demus, H. (1973) Subcellular Fractionation of Human Lymphocytes: Isolation of Two Plasma Membrane Fractions and Comparison of the Protein Components of the Various Lymphocytic Organelles. *Biochim. biophys. Acta*, **291**, 93.

De Pierre, J. W. & Karnovsky, M. L. (1973) Plasma Membranes of Mammalian Cells. A Review of Methods for Their Characterization and Isolation. *J. cell Biol.*, **56**, 275.

Garen, A. & Levinthal, C. (1960) A Fine-structure Genetic and Chemical Study of the Enzyme Alkaline Phosphatase of *E. Coli*. I. Purification and Characterization of Alkaline Phosphatase. *Biochim. biophys. Acta*, **38**, 470.

GATT, R. & BERMAN, E. R. (1966) A Rapid Procedure for the Estimation of Amino Sugars on a Micro Scale. *Anal. Biochem.*, **15**, 167.

GILES, K. W. & MYERS, A. (1965) An Improved Diphenylamine Method for the Estimation of Deoxyribonucleic Acid. *Nature, Lond.*, **206**, 93.

KIM, U. & PICKREN, J. W. (1974) Plasma Membrane-associated Enzymes in Metastasizing and Non-metastasizing Rat Mammary Carcinomas (MT). *Proc. Am. Ass. Cancer Res.*, **15**, 103.

KIM, U., BAUMLER, A., CARRUTHERS, C. & BIELAT, K. (1975) Immunological Escape Mechanism in Spontaneously Metastasizing Mammary Tumors. *Proc. natn. Acad. Sci. U.S.A.*, **72**, 1012.

LOWRY, O. H., ROSEBROUGH, N. J., FARR, A. L. & RANDALL, R. J. (1951) Protein Measurement with the Folin Phenol Reagent. *J. biol. Chem.*, **193**, 265.

MICHELL, R. H. & HAWTHORNE, J. N. (1965) The Site of Diphosphoinositide Synthesis in Rat Liver. *Biochim. biophys. Res. Commun.*, **21**, 333

PALADE, G. E. (1952) A Study of Fixation for Electron Microscopy. *J. exp. Med.*, **95**, 285.

PENNINGTON, R. J. (1961) Biochemistry of Dystrophic Muscle. Mitochondrial Succinate-tetrazolium Reductase and Adenosine Triphosphatase. *Biochem. J.*, **80**, 649.

PORTEOUS, J. W. & CLARK, B. (1965) The Isolation and Characterization of Subcellular Components of the Epithelial Cells of Rabbit Small Intestine. *Biochem. J.*, **96**, 159.

PROSPERO, T. D., BURGE, M. L. E., NORRIS, K. A., HINTON, R. H. & REID, E. (1972) Alkaline Ribonuclease and Phosphodiesterase Activity in Rat Liver Plasma Membranes. *Biochem. J.*, **132**, 449.

RAZZELL, W. E. (1961) Tissue and Intracellular Distribution of Two Phosphodiesterases. *J. biol. Chem.*, **236**, 3028.

ROE, J. H. (1955) The Determination of Sugar in Blood and Spinal Fluid with Anthrone Reagent. *J. biol. Chem.*, **212**, 335.

SCHLISELFELD, L. H., EYS, J. V. & TOUSTER, O. (1965) The Purification and Properties of a Nucleotide Pyrophosphatase of Rat Liver Nuclei. *J. biol. Chem.*, **240**, 811.

SCHNEIDER, W. C. (1957) Determination of Nucleic Acids in Tissues by Pentose Analysis. In *Methods of Enzymology*, Vol. 3. Eds. S. P. Colowick and N. Kaplan. New York: Academic Press.

SEARCY, R. L., BERGQUIST, L. M. & JUNG, R. C. (1960) Rapid Ultramicro Estimation of Serum Total Cholesterol. *J. lipid Res.*, **1**, 349.

SPURR, A. R. (1969) A Low-viscosity Epoxy Resin Embedding Medium for Electron Microscopy. *J. ultrastruc. Res.*, **26**, 31.

SWANSON, M. A. (1950) Phosphatases of Liver. I. Glucose-6-Phosphatase. *J. biol. Chem.*, **184**, 647.

TOUSTER, O., ARONSON, N. N. JR, DULANEY, J. T. & HENDRICKSON, H. (1970) Isolation of Rat Liver Plasma Membranes. Use of Nucleotide Pyrophosphatase and Phosphodiesterase I as Marker Enzymes. *J. cell Biol.*, **47**, 604.

TUNIS, M., KIM, U. & CARRUTHERS, C. (1973) Correlation of an Enzyme Profile with the Metastasizing Capacity of Rat Mammary Carcinomas. *Proc. Am. Ass. Cancer Res.*, **14**, 80.

WARREN, L. (1959) The Thiobarbituric Acid Assay of Sialic Acids. *J. biol. Chem.*, **234**, 1971.